# Wigner distribution of self-amplified spontaneous emission free-electron laser pulses and extracting its autocorrelation

**DOI:** 10.1107/S160057752001382X

**Published:** 2021-01-01

**Authors:** Svitozar Serkez, Oleg Gorobtsov, Daniel E. Rivas, Michael Meyer, Bohdana Sobko, Natalia Gerasimova, Naresh Kujala, Gianluca Geloni

**Affiliations:** a European XFEL, Hamburg, Germany; b Deutsches Elektronen-Synchrotron (DESY), Hamburg, Germany; c Lviv National University, Lviv, Ukraine

**Keywords:** FEL, Wigner, spectrogram, duration, SASE

## Abstract

The emerging concept of ‘beam by design’ in free-electron laser (FEL) accelerator physics aims for accurate manipulation of the electron beam to tailor spectral and temporal properties of the radiation for specific experimental purposes, such as X-ray pump/X-ray probe and multiple wavelength experiments. ‘Beam by design’ requires fast, efficient, and detailed feedback on the spectral and temporal properties of the generated X-ray radiation. Here a simple and cost-efficient method to extract information on the longitudinal Wigner distribution function of emitted FEL pulses is proposed. The method requires only an ensemble of measured FEL spectra and is rather robust with respect to accelerator fluctuations. The method is applied to both the simulated SASE spectra with known radiation properties as well as to the SASE spectra measured at the European XFEL revealing underlying non-linear chirp of the generated radiation. In the Appendices an intuitive understanding of time–frequency representations of chirped SASE radiation is provided.

## Abstract 2

An ensemble-averaged Wigner distribution is a useful tool for time–frequency analysis of simulated SASE FEL pulses. Here it is shown how to calculate its frequency-wise autocorrelation based on a set of measured single-shot SASE spectra.

## 1. Introduction   

Various fields of science such as structural biology (Seibert *et al.*, 2011 ▸; Chapman *et al.*, 2011 ▸), plasma physics (Vinko *et al.*, 2012 ▸), atomic physics (Young *et al.*, 2010 ▸), ultrafast photochemistry (Liekhus-Schmaltz *et al.*, 2015 ▸), and many others have benefited from the development of X-ray and extreme ultraviolet (XUV) free-electron lasers (XFELs) (Saldin *et al.*, 2010 ▸; McNeil & Thompson, 2010 ▸; Pellegrini *et al.*, 2016 ▸). FELs provide a unique combination of high power with narrow-bandwith, ultra-short pulses with tunable photon energy. For instance, at the European XFEL, powers of tens of GW with relative spectral width in the order of 10^−3^–10^−4^, ten fs-order duration and photon energies between 0.25 keV and 25 keV are within specifications.

There has been a rapid growth both in the number of scientific users and in the diversity of new science enabled by FEL sources (Hemsing *et al.*, 2014 ▸). This growth is possible due to the continuously improving capabilities of FEL facilities. One promising avenue to new experiments is tuning the spectral and temporal properties of radiation for specific experimental purposes, *e.g.* generating large-bandwidth (Serkez *et al.*, 2013 ▸; Zagorodnov *et al.*, 2016 ▸; Prat *et al.*, 2016 ▸), narrow-bandwidth (Reiche, 2013 ▸) or extremely short (Zholents, 2010 ▸; Serkez *et al.*, 2018 ▸) radiation pulses.

All these and other techniques require ‘beam by design’ (Hemsing *et al.*, 2014 ▸) via precise manipulation of the accelerator conditions and rely upon accurate diagnostics of electron beam and radiation pulse parameters. Of particular importance is the information on the longitudinal phase space of electrons or on the spectrogram (time–frequency representation) of the radiation. They can be obtained by installing a transverse deflecting structure (Behrens *et al.*, 2014 ▸) or an optical streaking setup (Grguraš *et al.*, 2012 ▸; Hartmann *et al.*, 2018 ▸), respectively. Both methods require the installation of additional complicated hardware downstream every undulator line.

However, some information about the duration of the typical radiation pulse, hence the length of the electron beam lasing window, can be extracted from the radiation spectra of an FEL operating in self-amplified spontaneous emission (SASE) mode. The temporal shape of FEL pulses can be deduced by comparing energy losses between lasing and non-lasing electron beams with transverse deflecting structure (Behrens *et al.*, 2014 ▸).

Taking advantage of the statistical properties of SASE radiation (Saldin *et al.*, 1998 ▸) and assuming a particular temporal profile, measurement of spectral spike width (Inubushi *et al.*, 2012 ▸) or spectral correlation analysis (Lutman *et al.*, 2012 ▸) provides an estimate of the average duration of the SASE FEL pulse assuming its temporal profile. Correlation functions of Gaussian and sinc shapes correspond to Gaussian and flat-top radiation pulse profiles, respectively. The close relation between the electron phase space and the radiation characteristics must be taken into account. For example, a chirp in the electron beam energy yields a chirp in radiation frequency (Krinsky & Huang, 2003 ▸), and affects the range of spectral coherence (Gorobtsov *et al.*, 2018 ▸), and hence the spectrum-based estimation of the SASE pulse duration.

The resolution, as well as the minimum group duration applicable to the spectrum-based methods, is several coherence lengths of the SASE radiation, *i.e.* the length of several temporal ‘spikes’.

In this contribution we show that by exploiting the full information contained in the correlation function it is possible to analyze the shape of the radiation pulse at each photon energy. We present a fast and efficient method to provide feedback on the temporal and spectral properties of FEL radiation, namely the measurement of the autocorrelation of an ensemble-averaged Wigner distribution of SASE FEL pulses. The method relies entirely on spectrometry of the generated pulses and does not require additional equipment. It thus allows for straightforward implementation at existing and future FEL facilities.

In the following sections we study an ensemble-averaged Wigner distribution of SASE FEL pulses and its temporal autocorrelation. We discuss how to acquire an autocorrelation of this distribution based on measured SASE spectra and what information such reconstruction reveals. We finally present results of numerical simulations performed with the code *GENESIS* (Reiche, 1999 ▸) and compare calculated Wigner distributions with evaluated reconstructions. We also present results of an experimental application of the algorithm at the European XFEL where information about a nonlinear frequency chirp is extracted.

## 2. Theory   

In this section we describe the theoretical background at the basis of our retrieval algorithm. We then proceed to the formulation of the Wigner distribution autocorrelation reconstruction algorithm.

### 2.1. Definitions and conventions   

Consider a scalar field *E*(*t*) in the time domain and its Fourier transform 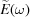,
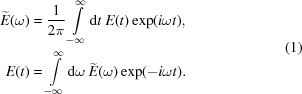
Measurable single-shot radiation spectra are proportional to the square-modulus of the single-shot scalar field^1^

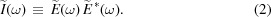
The statistical autocorrelation function of the field *E*(*t*) in the time and frequency domains can then be defined as
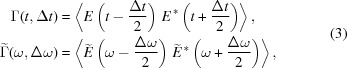
where angle brackets 〈…〉 denote the ensemble average. Note that the autocorrelation function depends on both time *t* and time separation Δ*t*, allowing to describe non-stationary radiation fields. The intensity autocorrelation function is, instead,
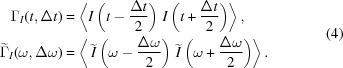
It is also customary to define and analyze normalized correlation functions (Saldin *et al.*, 1998 ▸). For example, in the frequency domain the normalized second-order correlation function is given by
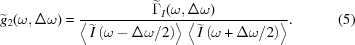
Two time–frequency representations widely used in signal processing are the spectrogram,
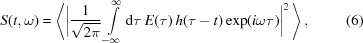
[here *h*(*t*) is a spectrogram window function] and the Wigner distribution,
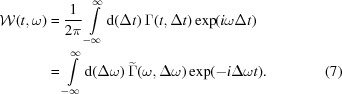
The latter is commonly used to describe properties of FEL radiation (Wu *et al.*, 2007 ▸; Allaria *et al.*, 2010 ▸; Marcus *et al.*, 2014 ▸; Huang *et al.*, 2016 ▸; Serkez, 2016 ▸).

The spectrogram of a signal *f*(*t*) can be expressed through the two-dimensional convolution of the Wigner distribution of that signal 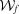 and the Wigner distribution 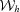 of the spectrogram window function *h*(*t*),
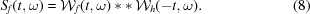
Here the symbol ** denotes a two-dimensional convolution operator. For more details see Appendix *A* [App appa] and Serkez *et al.* (2018 ▸).

The Wigner distribution is real but can take negative values, and yields the so-called ‘cross terms’ between signals in its time–frequency representation. Nevertheless, when averaged over an ensemble, it is positive for a great number of non-stationary processes which allows one to interpret it similarly to a radiation spectrogram (Flandrin, 1986 ▸). We find that this is also the case for the SASE FEL radiation, as shown in Appendix *B* [App appb].

In contrast to the Wigner distribution, the spectrogram is positive, but it fails to yield radiation and spectral powers via its marginal distributions, as they are convolved with window functions (see Fig. 14 in Appendix *B* [App appb]).

If the area covered by 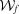 is much larger than that of 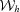 (namely, the radiation pulse is far from its transform limit, which applies to a typical SASE spectrum with large number of spikes), then, given a proper choice of a window function, the effect of convolution is small, the outlines of the statistically averaged Wigner and the spectrogram are similar and to a certain extent these distributions can be referred to interchangeably.

However, the calculation of both Wigner distribution and spectrogram relies on the knowledge of the *complex amplitude* of the pulse, while the experimentally measurable single-shot spectra are *intensity* distributions. This lack of phase information necessitates introduction of a different time–frequency representation.

### 2.2. Wigner distribution autocorrelation   

Let us assume that a scalar field *E*(*t*) obeys Gaussian statistics (Goodman, 2000 ▸) [which is the case for SASE FEL radiation strictly in the linear regime and approximately at saturation (Lutman *et al.*, 2012 ▸)]. Then, the moment theorem for Gaussian random variables can be applied to the intensity autocorrelation function (Mandel & Wolf, 1995 ▸, Section 8.4.1), to obtain
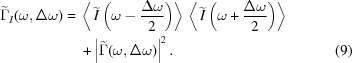
Let us now consider the following Fourier transform, 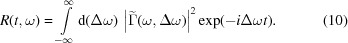
Using the autocorrelation theorem we can equate
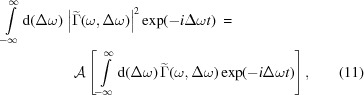
where 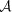 denotes the autocorrelation, defined as it is generally done in signal processing,^2^

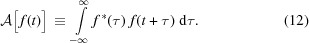
We now note that the argument of the autocorrelation product in equation (11) is the Wigner distribution function, and therefore
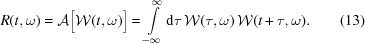

*R*(*t*, ω) is the frequency-wise temporal autocorrelation of the Wigner distribution and can be directly calculated based on measured spectra of SASE FEL radiation. We refer to this function as the reconstruction of spectrogram autocorrelation or, for short, ROSA.

One of its properties is that its marginal distribution on the frequency domain yields the square of the average radiation spectrum,
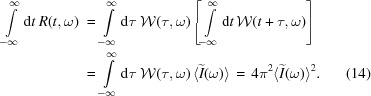
A cut *R*(*t*, ω = ω_0_) provides the autocorrelation of the intensity after bandpass filtering at frequency ω_0_,
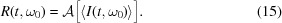
The integral of a reconstruction is roughly proportional to the square of the integral of a spectrogram,
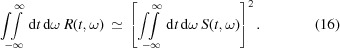



### 2.3. ROSA algorithm   

The algorithm of reconstruction of the spectrogram autocorrelation consists of the following conceptual steps.

First, sufficiently large statistics (around a thousand events) of single-shot SASE FEL spectra in the form of equation (2) are acquired. Here we assume that only SASE-related fluctuations are present. Otherwise, the measured data should be filtered since they are prone to additional jitter, unrelated to the SASE process, see Appendix *C* [App appc].

Second, we calculate the quantity
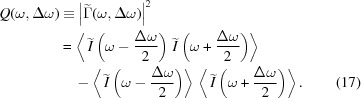
Finally, an inverse Fourier transform yields the reconstruction function *R*(*t*, ω),
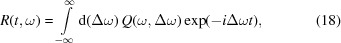
which is very similar in its formulation to the Wiener–Khinchin relation, see Appendix *D* [App appd].

We have found that binning of the reconstruction function *R*(*t*, ω) over several points in both dimensions greatly reduces numerical noise with practically no cost for effective resolution. This binning effectively serves as convolution of the Wigner distribution of the signal with that of a window function, as in equation (8), effectively yielding an autocorrelation of the radiation spectrogram.

### 2.4. Factorization for quasi-stationary pulses   

The expression for *R*(*t*, ω) is considerably simpler in an asymptomatic case of a quasi-stationary process. This is the particular case of constant instantaneous frequency and bandwidth over the FEL pulse. The assumption of quasi-stationarity allows us to neglect the dependence of the normalized correlation functions on the central frequency ω, *i.e.* they depend only on the frequency separation 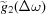.

Then one factorizes the autocorrelation functions as
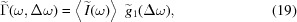


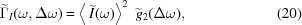
and, consequently, the reconstruction function
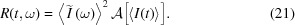
By integrating over frequencies, or fixing any value ω = ω_0_, we see that the reconstructed function is simply, aside from an unimportant multiplicative constant, the autocorrelation of the FEL pulse power in the time domain.

The autocorrelation of the flat-top power profile with length Δ*s* would yield an autocorrelation result with triangular shape and half width at half-maximum (HWHM) equal to Δ*s*/2. In the case of a Gaussian radiation pulse with FWHM Δ*s*, the HWHM size of its autocorrelation result will be 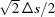. The standard deviation size of the autocorrelation is 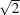 times larger than that of the original pulse independent of its shape, providing figure of merit independent of pulse shape.

## 3. Numerical simulations and discussions   

In order to illustrate the reconstruction capabilities of ROSA, we simulated four ensembles of FEL spectra with the simulation code *GENESIS* (Reiche, 1999 ▸), and processed them using the *OCELOT* package (Agapov *et al.*, 2014 ▸; Ocelot, 2020 ▸) to apply the ROSA algorithm.

For an input spectral data set we generated 500 statistically independent SASE events assuming a model 6 µm-long flat-top electron beam with (I) and without (II) energy chirp. In addition, we simulated SASE generation by two 2 µm-long flat-top electron beams separated by 3 µm (III). Finally, we computed 1000 statistically independent SASE events assuming a nominal 100 pC electron beam from start-to-end simulations for the European XFEL (IV) (Altarelli *et al.*, 2006 ▸; Zagorodnov, 2014 ▸).

The slice properties of the model beams are chosen to be close to those of the 100 pC nominal electron beam. The radiation generated by the model beams was dumped before saturation to reduce the radiation slippage and maintain the illustrative flat-top distributions of the ensemble-averaged radiation power. The realistic 100 pC beam radiation was dumped, instead, in deep saturation.

**Figure 1:**
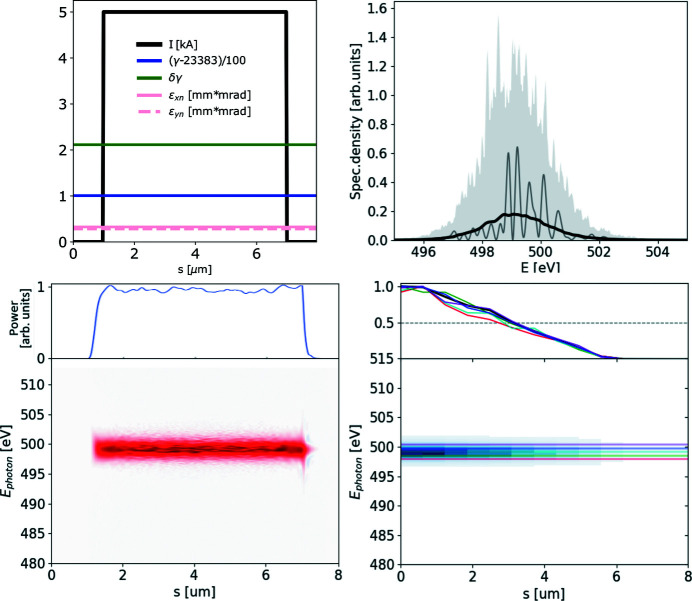
A 6 µm-long flat-top model electron beam without energy chirp, see top left plot, used to generate SASE radiation. Lines *I*, γ, δγ, ɛ_*xn*_, ɛ_*yn*_ depict electron beam current, energy (as Lorenz factor), energy spread and both horizontal and vertical normalized emittances, respectively. The radiation file is dumped during the exponential growth for 500 statistically independent events. SASE spectra are presented on the top right plot. The light gray area depicts spectral span of individual events, the dark gray line depicts a single event, and the black line provides the ensemble-averaged spectrum. The ensemble-averaged Wigner distribution of the SASE radiation is presented in the bottom left plot. Hereafter the diverging colormap of the Wigner distribution is normalized to its maximum absolute value, while its zero value is depicted with a white color. The spectrogram autocorrelation reconstruction *R*(*s*, ℏω/*e*) is presented in the bottom right plot. Colored lines in the top subplot show the corresponding line-offs of the reconstruction at different photon energies. These line-offs are normalized to 1 at their maximum value. The black line depicts an average of these line-offs. *s* = −*ct* is the coordinate along the radiation propagation direction.

The ROSA function (bottom right panels of Figs. 1 ▸, 2 ▸, 3 ▸, 4 ▸) for the simulated radiation is calculated with equation (18). The coordinate along the propagation direction *s* = −*ct* is related to the radiation arrival time. The reconstruction is symmetrical with respect to the *s* coordinate, hence only half of it is depicted. The ensemble-averaged Wigner distribution of the radiation (bottom left in Figs. 1 ▸, 2 ▸, 3 ▸, 4 ▸) is based on information about amplitudes and phases of the SASE radiation provided by the simulation to assert representativeness of the bottom right plot.

If no energy chirp is present in the electron beam, the undulator resonance condition is constant along the beam and the generated radiation pulse has no frequency chirp (Fig. 1 ▸). In this special case the Wigner distribution, and hence the reconstruction, are factorisable [equation (21)] and the total pulse length can be estimated.

**Figure 2:**
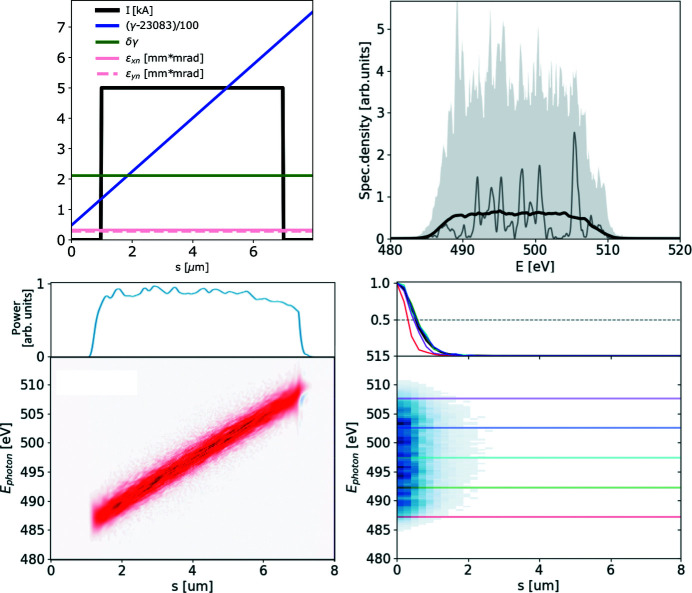
A 6 µm-long flat-top model electron beam with linear energy chirp, see top left plot, used to generate SASE radiation. It is dumped during the exponential growth for 500 statistically independent events. Subplots and notations are identical to those in Fig. 1 ▸.

When the energy chirp in the electron beam, in terms of the relative difference of electron energy in the head and tail, becomes comparable with the FEL efficiency parameter Δγ/γ ≳ ρ, a frequency chirp along the SASE pulse will appear. As a consequence, the pulse will yield a broader spectrum (which is the integral of the Wigner distribution over time) and typically a shorter pulse length at each photon energy (Krinsky & Huang, 2003 ▸) (horizontal line-offs of the Wigner distribution), as presented on Fig. 2 ▸. These effects are also reflected in the spectral correlation functions, and, if not accounted for, an underestimation of the total pulse duration will take place. Spectrum-based reconstructions cannot provide information on the group delay of different photon energies, as this information is lost with radiation phases.^3^

**Figure 3:**
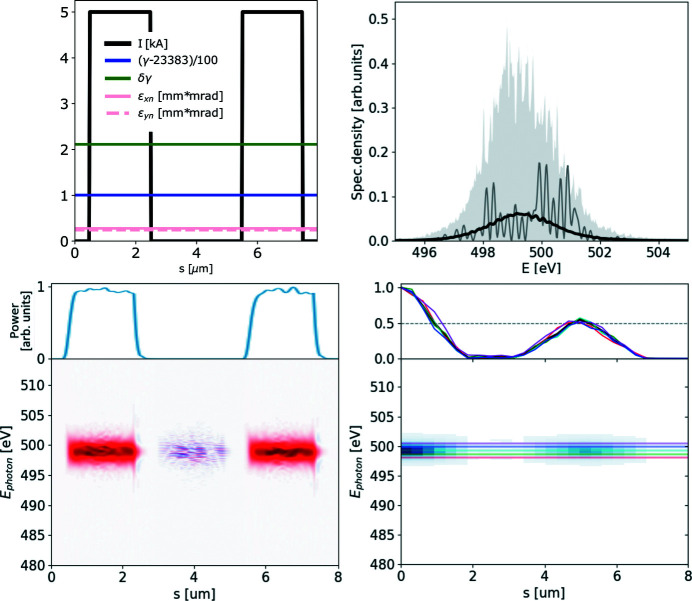
Two flat-top 2 µm-long electron beams, separated by 3 µm, generate two consecutive SASE pulses of the same averaged shape – see top left plot. The radiation is dumped during the exponential growth for 500 statistically independent events. Subplots and notations are identical to those in Fig. 1 ▸.

Two sequential SASE pulses with overlapping spectra, separated by time Δ*t*, will yield a combined spectrum with modulated amplitude, on top of the typical SASE modulation. The modulation period is given by
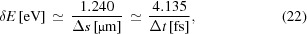
where Δ*s* and Δ*t* are the pulse separations in space (micrometres) and time (femtoseconds), respectively. If a spectrometer is capable of resolving this modulation, one can estimate the temporal separation of the pulses. This scenario is exemplified on Fig. 3 ▸. The discussed modulation of spectral density takes place only at the frequencies common for both pulses; therefore if individual spectra of the two pulses do not have common frequencies, *i.e.* do not overlap in the frequency domain, no ‘beating’ in this domain will take place.

Note that in the example above both the Wigner distribution and its reconstruction indicate the same temporal separation between the SASE pulses at all photon energies. If the temporal separation between the pulses is larger than their duration, the average ratio between the energies of the pulses *R*_*E*_ within certain bandwidth can be retrieved as 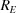 = 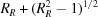, where *R*_*R*_ is the ratio of the integrals under first and second maximum in the reconstruction within the same bandwidth. In other words, the ratio of the areas under line-offs in reconstruction allows the ratio of energies of the investigated pulses to be calculated.

**Figure 4:**
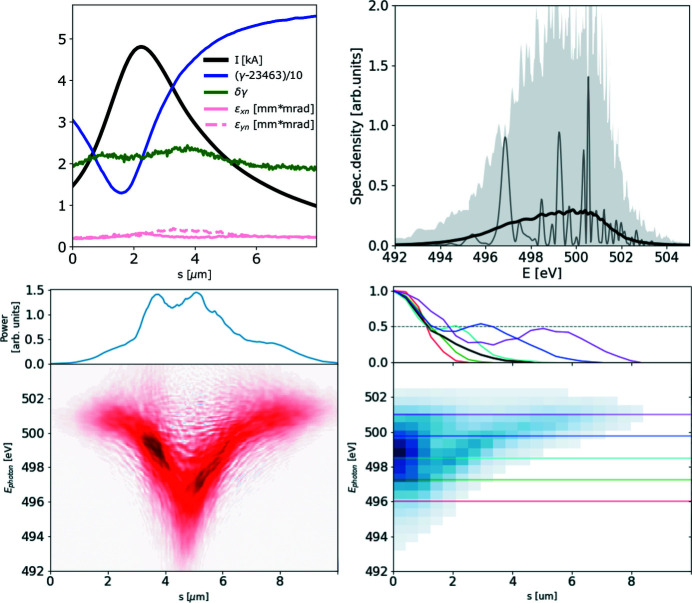
The European XFEL 100 pC electron beam with a non-linear energy chirp produces SASE radiation with different durations at different photon energies. Note the bifurcation in Wigner distribution above 499 eV. Analysis based on 1000 simulated SASE spectra. Subplots and notations are identical to those in Fig. 1 ▸.

**Figure 5:**
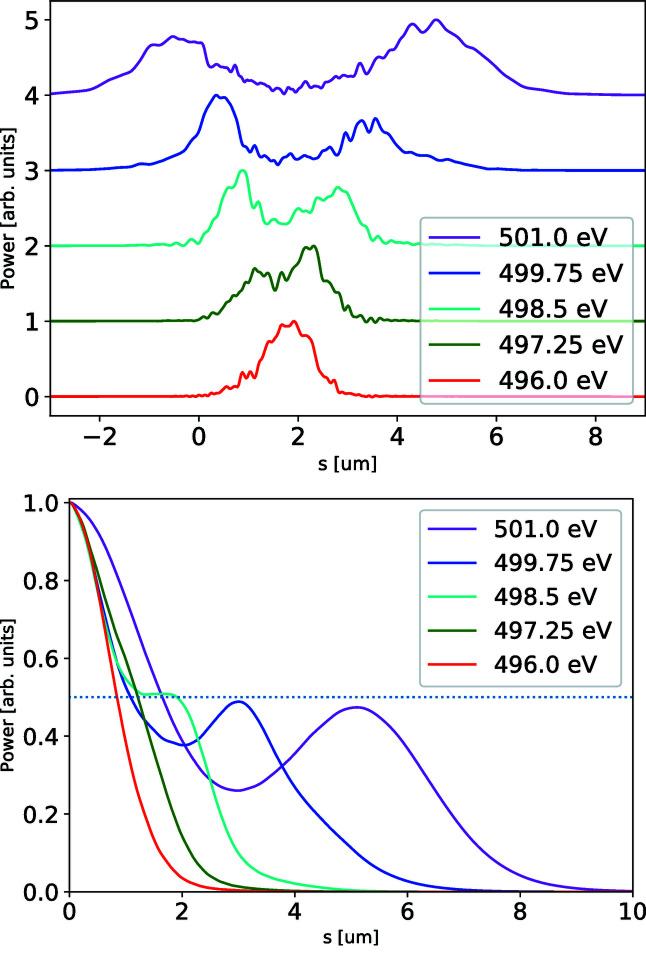
The line-offs of the Wigner distribution presented in Fig. 4 ▸ for different photon energies each binned over 0.2 eV (top plot) and their corresponding autocorrelation traces (bottom plot). The color convention follows that of the line-offs of the reconstruction on the same figure.

In general, the electron beam formation system may yield a highly non-linear energy chirp, as illustrated in Fig. 4 ▸ (top left plot). If the relative peak-to-valley energy difference in the electron beam is comparable with or larger than the Pierce parameter ρ, the electron beam energy chirp will be imprinted into the SASE radiation spectrogram as a radiation frequency chirp. In the given example the Wigner distribution at 501 eV yields two distinct pulses separated by about 5.5 µm (18 fs), as depicted in Fig. 5 ▸. The separation of these sub-pulses at given frequency grows with the photon energy, following the separation of the electron beam slices with an equal Lorenz factor γ. Similarly to the double-pulse case, illustrated in Fig. 3 ▸, such photon-energy-dependent separation can be straightforwardly observed in the reconstruction function.

Therefore the proposed reconstruction method may help to diagnose non-linear energy chirps in the electron beam. It also allows to estimate the duration and temporal shape of the SASE radiation upon spectral filtering. We show the robustness of the proposed method in Appendix *C* [App appc]

## 4. Experimental results   

In order to assess the actual performance of our technique, we studied a set of 700 single-shot SASE spectra acquired with the SASE3 beamline spectrometer (Gerasimova, 2018 ▸). SASE radiation at 495 eV photon energy was emitted in the SASE3 undulator line with a 250 pC electron beam, accelerated to 11.5 GeV. All 21 undulator segments were closed and quadratically tapered starting from cell 7.^4^ Each undulator segment is 5 m long with 68 mm periods. The spectrometer resolution was not characterized for the given YAG imager with which the spectra were obtained; however, the experimental data suggest that the effective resolution of the acquired data is better than 0.15 eV FWHM.

**Figure 6:**
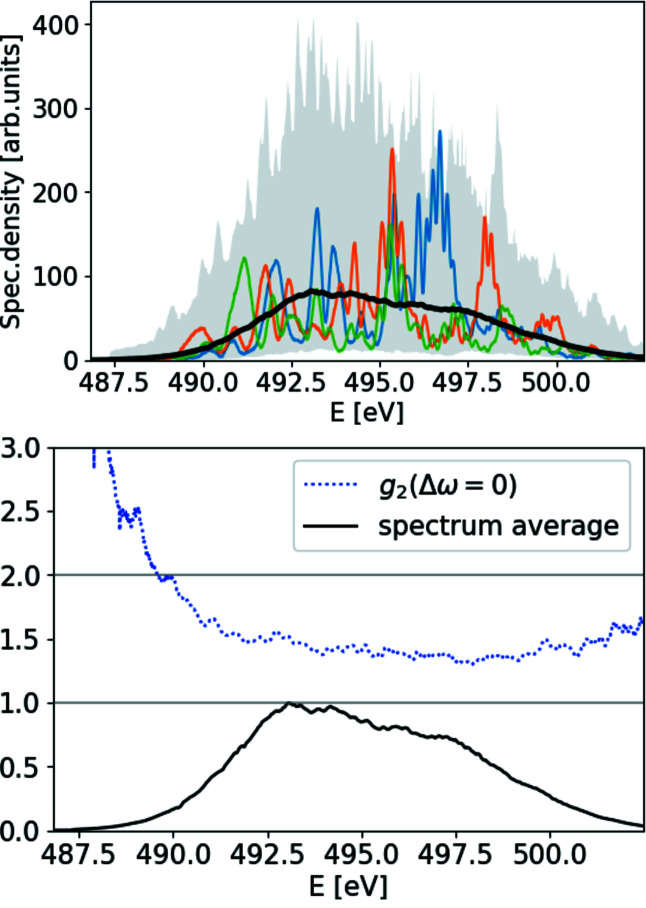
Top: ensemble average of measured soft X-ray SASE spectra (solid black line), spectral density range within the ensemble (gray background area) and three single-shot measurements of spectra (green, blue and orange lines). Bottom: value of normalized second-order correlation function at Δω = 0.

**Figure 7:**
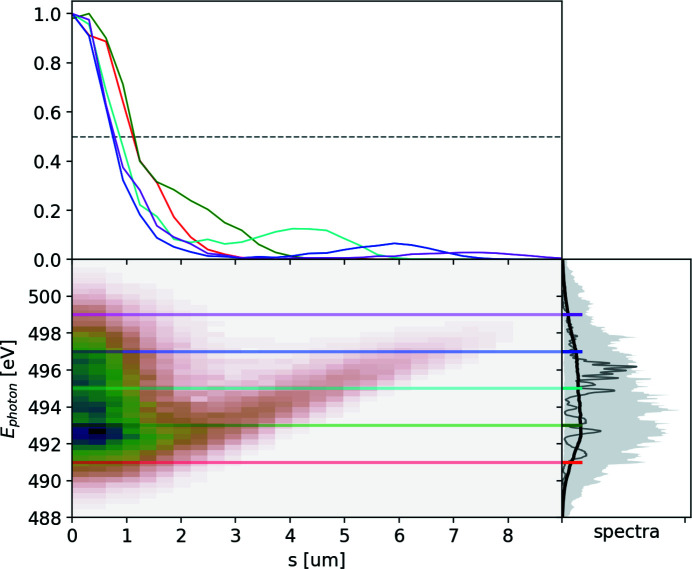
Result of processing the soft X-ray experimental spectra with the ROSA algorithm.

The resulting spectra and the quality factor are provided in Fig. 6 ▸, and the corresponding ROSA result is depicted in Fig. 7 ▸. In the central region of the spectrum the quality factor deviates from the value of 2 and we attribute it to the limited resolving power of the spectrometer and poor spatial coherence of the FEL radiation, as actually expected in deep saturation and to a photon energy jitter/drift, see Fig. 18.

The reconstruction and its line-offs are nevertheless informative: there is a clearly visible, characteristic divergent structure with length of about 6 µm, qualitatively similar to that discussed in Fig. 4 ▸.^5^ It suggests the presence of two radiation sub-pulses with photon-energy-dependent separation at a rate of about 1.5 µm eV^−1^. Thereby ROSA indicates the presence of quadratic frequency chirp in the emitted radiation. The quadratic component of the detected chirp, as expected, results in lower instantaneous radiation frequency in the middle of the pulse. From the reconstruction we can deduce two sub-pulses at the 497 eV photon energy, separated by about 6 µm (20 fs). The ratio of integrals under the corresponding line-off indicates the pulse energy ratio below 9.5:1 at that separation, making it our lower estimate on the total pulse duration span.

One reason the total pulse may be longer is that at the photon energies above 497 eV no second sub-pulse is present because of an asymmetric shape of the radiation spectrogram. Another possible reason is due to a natural limit of the reconstruction time window from the effective resolving power of the spectrometer, discussed in the previous section and illustrated in Fig. 17. The latter ‘dampens’ the output of ROSA at larger time scales.

The theoretical resolving power of the SASE3 spectrometer at 500 eV is of the order of *R* ≃ 6000 (resolution < 0.09 eV) and can be improved by operating the grating at the second diffraction order with *R* ≃ 12000.

**Figure 8:**
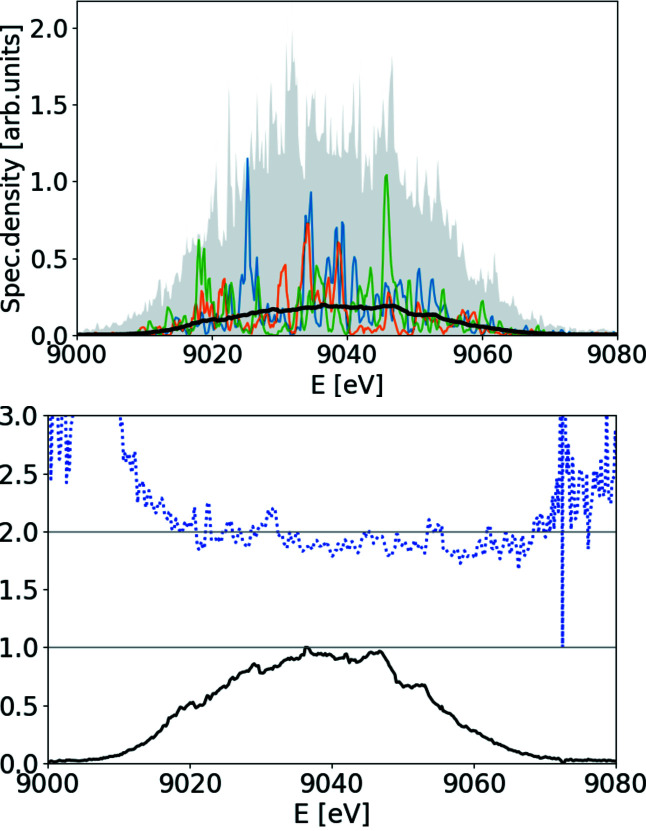
Top: ensemble average of measured hard X-ray SASE spectra (solid black line), spectral density range within the ensemble (gray background area) and three single-shot measurements of spectra (green, blue and orange lines). Bottom: value of normalized second-order correlation function at Δω = 0.

**Figure 9:**
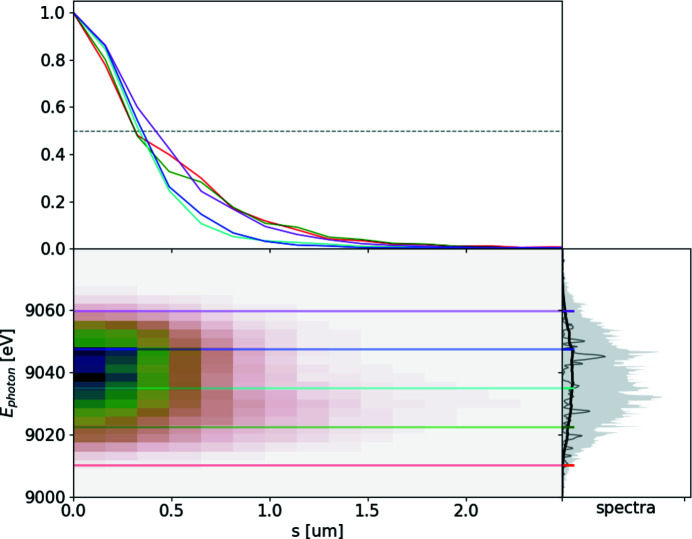
Result of processing the hard X-ray experimental spectra with the ROSA algorithm.

**Figure 14:**
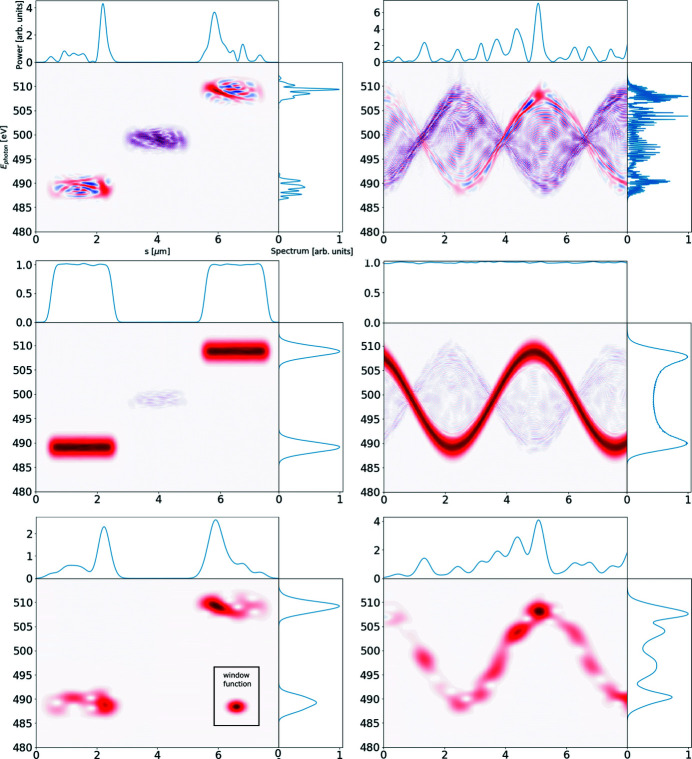
Wigner distribution of imitated SASE FEL radiation for two ‘flat-top’ pulses with different frequencies (left subfigures) and for a continuous pulse with instantaneous frequency varying in time sinusoidally (right subfigures). The distributions averaged over an ensemble of one and 10000 statistically independent realizations are presented on the top and middle subfigures, respectively. The amplitude of the cross terms is reduced significantly upon averaging over an ensemble. The bottom subfigures illustrate the single-shot spectrograms – the result of Wigner distribution convolution with that of a window function, provided in the inset.

**Figure 17:**
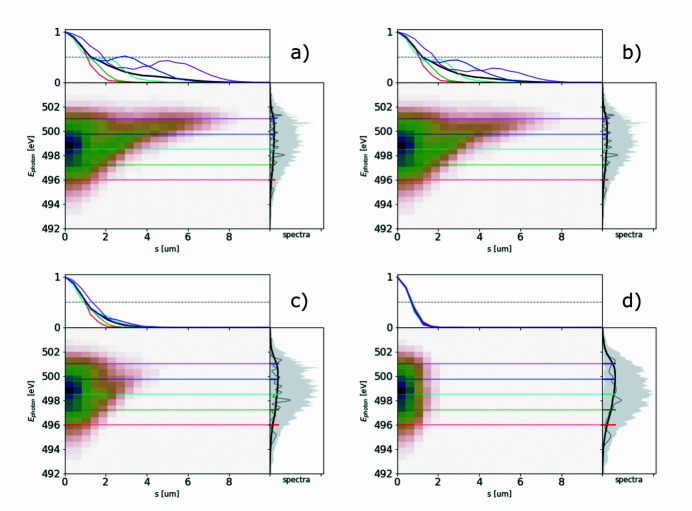
Spectrogram autocorrelation function upon simulating limited spectrometer resolution imitated by convolving spectra with Gaussian instrumental function of (*a*) 0.03 eV, (*b*) 0.07 eV, (*c*) 0.2 eV and (*d*) 0.5 eV FWHM bandwidth.

**Figure 18:**
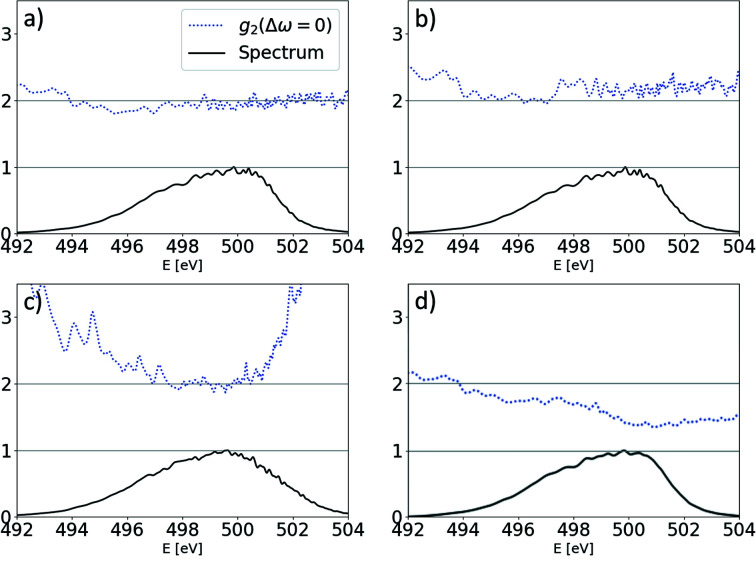
Averaged spectrum with corresponding values of 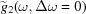 in the case of (*a*) no detrimental effects introduced, (*b*) added 100% pulse energy jitter, (*c*) 1 eV photon energy jitter and (*d*) convolving spectra with 0.2 eV FWHM instrumental function.

We applied ROSA to a series of hard X-ray spectra, depicted in Fig. 8 ▸. It was measured at the SASE2 beamline with the High Resolution X-ray Spectrometer HIREX (Grünert *et al.*, 2019 ▸; Kujala *et al.*, 2020 ▸), equipped with a one-dimensional high-repetition-rate Gotthard detector. The SASE radiation was dispersed using a bend silicon crystal with 440 reflection. At the time of the data acquisition the energy of the electron beam was chirped. This increased the radiation bandwidth to 0.5% from the expected value of about 0.15%. This chirp affected the group duration of the radiation, which is detectable by ROSA or other spectral measurement methods. The FWHM of the group duration in the middle of the spectrum at 9035 eV is about 2 fs (see Fig. 9 ▸).

The HIREX spectrometer in given configuration can provide a resolution of 0.2 eV. It can be decreased to 0.09 eV by employing a two-dimentional 10 Hz Photonic Science camera.

With a spectral resolution below 0.1 eV both SASE3 and SASE2 spectrometers allow us to study SASE pulses with group durations up to 16 fs providing important diagnostics for the short pulse operation mode.

We can conclude that the numerical simulations provide a good estimate for the behavior of ROSA, and that the ROSA output from experimental data is relatively easy to interpret and is consistent with the simulation results with start-to-end electron beam.

## 5. Conclusions   

An ensemble-averaged Wigner distribution is useful for visualizing the time–frequency properties of numerically simulated SASE radiation when full information on the electric field distribution is available.

While the electric field distribution cannot be easily measured experimentally, one can reconstruct the *frequency-wise temporal autocorrelation* of the ensemble-averaged Wigner distribution of the radiation based on an experimentally measureable ensemble of spectra that enable calculations of the second-order spectral correlation function. The reconstructed Wigner function autocorrelation, upon noise filtering via binning, is close in terms of its properties to the well known spectrogram distribution. We call the proposed reconstruction method ROSA, reconstruction of spectrogram autocorrelation.

It constitutes an extended method to study the ensemble-averaged time–frequency distribution of relatively short X-ray SASE FEL pulses. The method does not require any hardware aside from a high-resolution single-shot spectrometer, which is typically available at XFEL facilities. The spectrometer resolution limits the maximum group duration of the pulse that can be analyzed.

The proposed method allows characterizing the pulse group duration and the approximate temporal shape individually for any photon energy present in the radiation. For instance, it indicates the presence of two temporally separated FEL pulses with common photon energies and provides information about their duration and temporal separation. In simulations, comparison of calculated Wigner distributions with the respective ROSA distributions shows that the method provides extensive information about the pulse structure.

ROSA relies on the fact that FEL pulses are short, narrow-bandwidth, and follow Gaussian statistics. It is statistical in nature and is based on the assumption that FEL hardware provides a reproducible electron beam moving along a stable orbit. Otherwise, discrimination of outlier events should take place.

In comparison with the conventional method of fitting the second-order spectral correlation function with a theoretical form-factor (Lutman *et al.*, 2012 ▸), ROSA does not require an initial assumption on the power profile of the SASE pulse. On the contrary, it yields additional information about the time–frequency distribution of SASE radiation. By exploiting the full information contained in the correlation function it is possible to analyze the shape of radiation pulse at each photon energy which allows, for instance, to diagnose the presence of sub-pulses at each photon energy.

We applied ROSA to an ensemble of SASE spectra measured at both SASE3 and SASE2 undulator lines at the European XFEL. We were able to diagnose the presence of a strong quadratic and linear frequency chirps in the X-ray pulses. In addition, the method provided a lower estimate on the total pulse duration. Due to this, ROSA may facilitate the analysis of experimental results, radiation diagnostics while performing a certain class of pump–probe experiments with small delay and can be used as an objective function to provide slow feedback to the accelerator.

The minimum pulse duration measureable by spectrum-based methods is limited to several coherence lengths, which almost certainly would exceed the temporal resolution of a transverse deflecting structure, in particular at high electron energies, making the two approaches complementary.

Our results suggest that the analysis of ROSA is more informative than fitting of the 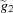 function, especially in the presence of non-linear frequency chirps. Due to the successful application to the real experimental data we thus conclude that ROSA may be a valuable tool for diagnostics of XFEL operation and ‘beam-by-design’ applications.

## Figures and Tables

**Figure 10:**
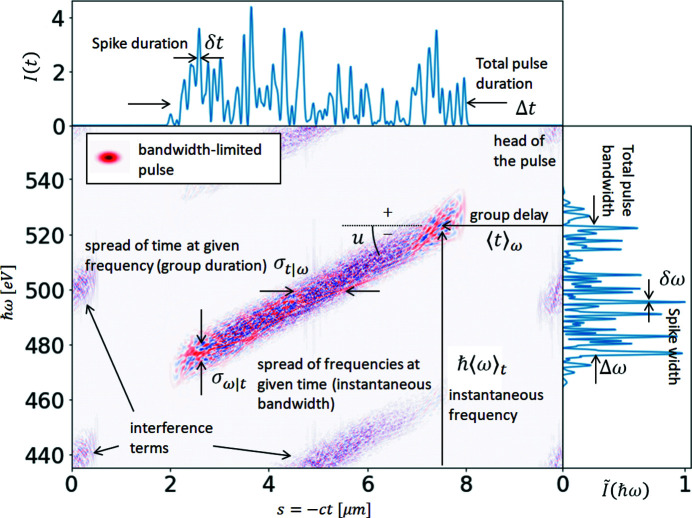
Time–frequency analysis terminology illustrated on a single-shot Wigner distribution of a modeled SASE pulse with negative frequency chirp *u*. The Wigner distribution for a bandwidth-limited pulse of Gaussian shape is provided in the inset.

**Figure 11:**
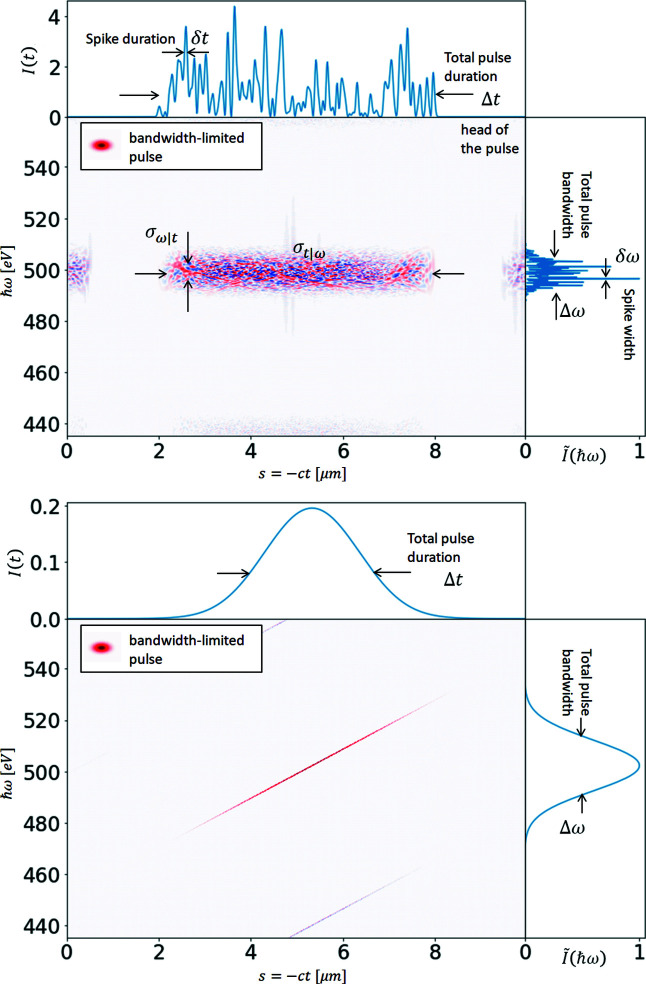
Single-shot Wigner distributions for, otherwise temporally coherent, Gaussian pulse with identical chirp and the same SASE pulse as in Fig. 10 ▸ without frequency chirp.

**Figure 12:**
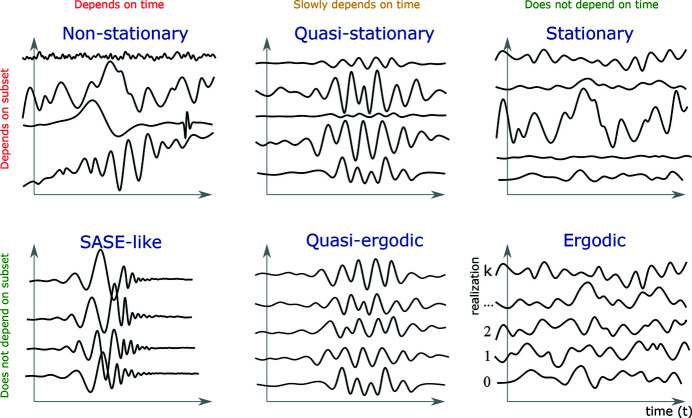
Illustration of statistical processes arranged according to whether their average depends on the choice of realization subset or the shift of time.

**Figure 13:**
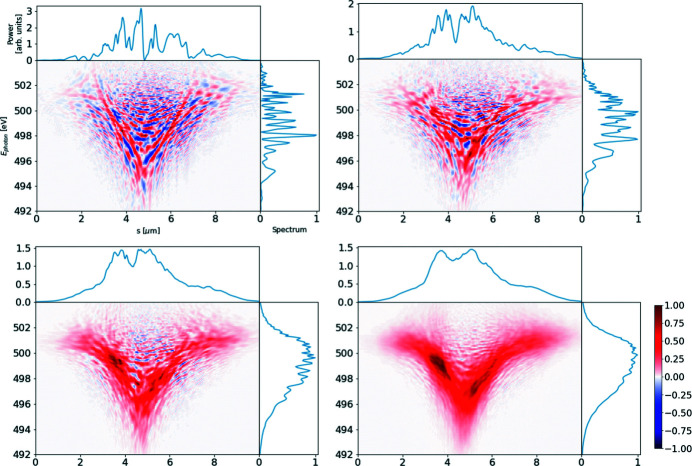
Colormap representations of the Wigner distribution of simulated SASE FEL radiation with their marginal distributions when averaged over an ensemble of 1 (upper left subfigure), 10 (upper right), 100 (lower left) and 1000 (lower right) statistically independent realizations. Note the significant non-linear frequency chirp in the pulse, visible upon ensemble averaging.

**Figure 15:**
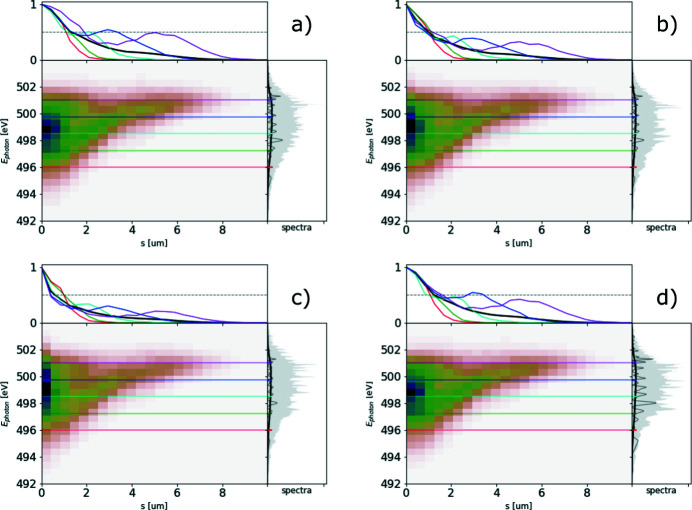
Spectrogram autocorrelation function upon adding the pulse energy jitter of (*a*) 0%, (*b*) 50%, (*c*) 100% and (*d*) 100% after pedestal subtraction in the correlation function. Hereinafter, colored lines in the top subplots show the corresponding line-offs of the reconstruction at different photon energies.

**Figure 16:**
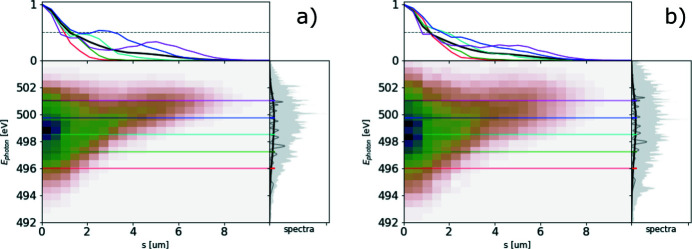
Spectrogram autocorrelation function upon adding the electron energy jitter corresponding to jitter of radiation spectra with r.m.s. of (*a*) 0.5 eV and (*b*) 1 eV.

## Footnotes

1 Considering the cross section perpendicular to the direction of propagation.

2 The statistical definition of the autocorrelation function is different.

3 Note that since the energy chirp increases the spectral bandwidth, a comparison with the expected bandwidth (*e.g.* using the results of numerical simulations for given electron beam properties and undulator settings) can give an estimate of the chirp. Nevertheless, the results would be based on *a priori* information, and cannot be conclusive, as in any method relying purely on spectral measurements.

4 Such operation mode with all undulators in resonance is not nominal for the SASE3 beamline. The emitted radiation is expected to have worse than usual transverse coherence.

5 Note that the calculations to illustrate the effect of a quadratic chirp (Fig. 4 ▸) precede the experimental results (Fig. 7 ▸), and no *quantitative* agreement between the two is expected.

## Appendix A Time–frequency representations and Wigner distribution

In order to characterize radiation pulses in both the temporal and the frequency domain, we consider a generalized class of time–frequency signal representations that was introduced by Cohen (Cohen, 1966 ▸, 1995 ▸),

where ϕ(Δω, Δ*t*) is a two-dimensional function called kernel (Claasen & Mecklenbrauker, 1980 ▸) and determines the particular representation in the class.

The Wigner distribution is one of the time–frequency representations with kernel ϕ(Δω, Δ*t*) = 1, useful to describe properties of FEL radiation,

Any representation of Cohen class can be expressed in terms of the Wigner distribution,

where

is just a different representation of Cohen kernel via its two-dimensional Fourier transform. The symbol ** denotes a two-dimensional convolution operator.

The spectrogram is another and much more known Cohen class function that facilitates time–frequency analysis,

It requires introduction of a window function *h*(*t*) in the time and frequency domain,

The kernel function of the spectrogram is more complicated,

From equations (25) and (26) it follows that the spectrogram of the signal *f* can be obtained by convolving the Wigner distribution *W*_*f*_ of that signal with the Wigner distribution *W*_*h*_ of the spectrogram window function *h*,

in other words the spectrogram is, in a way, a ‘smeared’ version of the Wigner distribution. In contrast to a Wigner distribution, a spectrogram is non-negative everywhere, but it fails to yield radiation and spectral powers via its marginal distributions, as they are also ‘smeared’ by being convolved with window functions,

Marginal distributions of the Wigner function (projections on time and frequency domains) are the radiation power and spectral power, respectively,

Its first conditional moments of time and frequency are the instantaneous frequency and group delay,

The conditional spreads of the Wigner distribution – local averages in frequency and time – can also be introduced as instantaneous bandwidth σ_ω|*t*_ and group duration σ_*t*|ω_,

alas, they may become negative, and hence cannot always be properly interpreted if no ensemble averaging was carried out (Cohen, 1995 ▸, p. 120).

A Wigner distribution of a single chirped SASE pulse with marginal distributions, first and second moments is illustrated in Fig. 10 ▸. The manifestation of the Wigner distribution interference terms is also visible as spots around the pulse without marginal distributions.

For comparison, Wigner distributions of the same pulse without chirp (left subfigure) and a coherent pulse with a Gaussian profile and identical chirp (right subfigure) are provided in Fig. 11 ▸.

It is worth noting that the instantaneous bandwidth of the SASE radiation is basically the FEL amplifier bandwidth determined by the electron beam properties and undulator parameters. It relates to the coherence time – temporal spike duration – at saturation as

As discussed by Krinsky & Huang (2003 ▸), in the presence of the frequency chirp *u* = Δω/Δ*t* in a sufficiently long pulse, the group duration is related to the instantaneous bandwidth as

so the spectral coherence range, and hence the spectrum spike width at certain frequency, is determined by the effective duration of the pulse with given frequency components,

One can see that only a group duration, not the entire pulse duration, is imprinted in SASE spectra and can be extracted by analysis of the spectral correlation function, unless additional information on the pulse chirp is available.

## Appendix B Stationarity, ergodicity and SASE radiation

The generation of SASE pulses starts the shot noise in the electron beam and yields quasi-monochromatic radiation (Saldin *et al.*, 2010 ▸). From a mathematical standpoint, the electric field in the pulse can be described as a random process.

Let us consider a random (complex scalar) process *a*. We denote with ^*k*^ *a*(*t*) the *k*th realization of the process, which is a function of the time *t*. If one fixes *t* = *t*_*m*_, *a*(*t*_*m*_) is a random variable, and ^*k*^ *a*(*t*_*m*_) one of its realizations, in our case a complex number. We introduce the ensemble average of the process *a* as

where the limit for a large number of samples  is usually taken. We define the time average of the *k*th realization of *a*(*t*) over the finite time interval *T* as

The full time-average of ^*k*^ *a* is defined in a natural way as

The process *a* is fully determined whenever we give the joint probability distributions

for all integer values *m*. For any given *m*, *p*_*a*,*m*_ is the probability density of finding, upon measurement of a generic realization, the complex values *a*_1_,…, *a*_*m*_ at times *t*_1_,…, *t*_*m*_, respectively. This specification allows calculating joint moments. For example, for the case for *m* = 1, which gives the ensemble average, one has

while one of the joint moments for *m* = 2 gives the first-order temporal correlation function

In the following we find it useful to describe the times *t*_1_,…, *t*_*m*_ as , that is in terms of a common time  and dispacements δ*t*_1_,…, δ*t*_*m*_, and changing likewise the integration variables in the various joint moments. This means that, in general,  = ). Note that here we introduced one extra degree of freedom in the definitions that can be used to adjust notations. For example, for *m* = 2 we have  = ,  =  and we can decide to set δ*t*_1_ = 0, δ*t*_2_ = Δ*t*, or δ*t*_1_ = −Δ*t*/2, δ*t*_2_ = Δ*t*/2, a choice to be made depending on notational convenience. In this section we will set δ*t*_1_ ≡ 0 so that  =  .

We recall that stochastic processes can be separated into several broad classes (Mandel & Wolf, 1995 ▸; Goodman, 2000 ▸). A *strict-sense stationary* random process is defined by the requirement that any joint probability distribution does not depend on . This means that  =  =    for any value of *m*. In a more narrowly defined sense, we can require stationarity only up to the *j*th order, requiring that only the joint moments up to *m* = *j* are independent of . In the case *j* = 2, the process is called *wide-sense stationary*, and the moments  and  are independent of . It is also straightforward to define a subclass of strict-sense stationary processes, known as *ergodic*, requiring that any average calculated statistically over the ensemble must be equal to the same average calculated over any fixed *k*th realization in time. For example, for *m* = 2,

From these definitions it follows that any radiation field described by a stationary random process has no beginning nor end, which strictly speaking is not physical. To fix this issue, we can introduce the concept of quasi-stationarity (in strict or wide sense) as well as of quasi-ergodicity by relaxing the condition of independence from  to a ‘slow’ dependence. The definition of the heuristic term ‘slow’ can be made more precise in terms of derivatives of probability density functions. Given  =  we require that

Since here we are particularly interested in the case *m* = 2, the condition above codifies a slowly varying dependence on  compared with the coherence time of the pulse, related to ‘how fast’ *p*_*a*,*m*_ changes with respect to δ*t*_2_ = Δ*t*. An important property of *quasi*-ergodic processes is that their ensemble-averaged temporal correlation function can factorize in factors depending separately on Δ*t* and , with a slow dependence on  with respect to Δ*t* and the coherence time of the pulse. Similarly, the Wigner distribution can be factorized in factors depending on ω and , *i.e.*  = . The Wigner distribution is therefore non-negative (Flandrin, 1986 ▸), which is extremely useful for a time–frequency analysis (Janssen & Claasen, 1985 ▸), because it allows one to interpret it as a positive probability distribution in a classical phase-space.

Strictly speaking, SASE FEL radiation in terms of its electric field as a function of time in the time domain or as a function of frequency in the frequency domain can be categorized within the hierarchy discussed above as a non-stationary process.^6^ However, SASE FEL radiation exhibits an important property that distinguishes it from fully random non-stationary processes. Since ensemble-averaged radiation intensity, coherence and instantaneous frequency depend on the electron beam properties (beam current, emittance, average electron energy within coherence time, *etc.*) and since the electron beam in FELs is usually reproducible on a shot-to-shot basis within the ensemble of data, the only random process involved in the generation of the SASE pulse is the intrinsic shot noise in the electron beam. This means that the various statistical averages of radiation quantities, and in particular the Wigner distribution, represent the properties of a typical radiation pulse upon convergence over any chosen subset of the entire ensemble. For instance, Wigner distributions, averaged over the events 1 to *k* and that averaged over the events *k* + 1 to 2*k* will be the same at large *k*. The situation is illustrated in Fig. 12 ▸ (lower left plot), which depicts the case of a SASE pulse duration comparable with the coherence time. The temporal correlation function (and the Wigner function) can no longer be factorized, in factors depending on Δ*t* and  separately, but at variance with Fig. 12 ▸ (upper left plot) the dependence on  is independent of the subset of the ensemble taken when performing statistical averages. Then, in the limit for a large number of longitudinal statistically independent modes, and of a slower and slower variation of the electron beam parameters over the number of these modes, the process becomes quasi-ergodic according to our previous definition.

With examples from Flandrin (1986 ▸) in mind, we find empirically that the ensemble-averaged Wigner distribution of SASE FEL radiation pulses converges towards non-negative values (for an increasing number of realizations in ensemble) even when the Wigner distribution is not separable and not delineated in the time–frequency representation plane. This convergence is illustrated in Figs. 13 ▸ and 14 ▸ where we present Wigner distributions of SASE FEL pulses, simulated with *GENESIS* code (Reiche, 1999 ▸) and of their imitation, modeled with an algorithm presented by Pfeifer *et al.* (2010 ▸). In both cases the radiation has statistical properties of a Gaussian random process (Mandel & Wolf, 1995 ▸, Section 3.1.4). Postprocessing is carried out using the *OCELOT* package (Ocelot, 2020 ▸).

## Appendix C ROSA applicability and robustness

In the derivation of the ROSA algorithm, we made certain assumptions about the radiation properties. Below we will discuss how the deviation of the pulse parameters in the ensemble affects the reconstruction results.

(i) Generally, accelerator diagnostics should be used to filter drifts and jitters of the radiation properties that are not related to the SASE process. Otherwise, long-range spectral correlations beyond the spectral spike width will appear and manifest themselves as a constant pedestal under the non-normalized second-order correlation function . This pedestal upon Fourier transform will yield a pronounced peak at *R*(*t* = 0,ω), as depicted in Fig. 15 ▸(*c*). However, by subtracting the pedestal in , one can recover a jitter-free reconstruction, see Fig. 15 ▸(*d*).

(ii) Significant electron energy jitter will cause jitter of the spectrum position. If it is not accounted for, the reconstruction *R*(*t*,ω) will be effectively smeared along the ω direction, as illustrated in Fig. 16 ▸.

(iii) The resolution of the spectrometer should be sufficient to visualize the pulse structure on the time scales of interest. Otherwise an underestimation of the group duration will take place, as illustrated in Fig. 17 ▸. Empirically we find that, in order to study pulses with group durations of σ_*t*|ω_, the resolution of the spectrometer in eV should ideally be better than 1.5/σ_*t*|ω_ [fs] FWHM. Decrease in the transverse coherence of the radiation pulse will have a similar effect, as it also affects fluctuations of the radiation power (Saldin *et al.*, 2010 ▸). The effect of limited coherence in a plane orthogonal to the dispersive direction can be mitigated by reducing the size of the spectrometer image integration region.

(iv) The spectrometer output should be representative of the actual spectrum and not be affected, for instance, by the *spatial* chirp of the SASE radiation (*i.e.* the head of the electron beam emits in a different direction than the tail). If such a chirp is present, it can be diagnosed by introducing apertures upstream of the spectrometer.

Owing to the properties of Gaussian statistics [see equation (9)], the value of the normalized second-order correlation function defined in equation (5), , at Δω = 0 should be ideally equal to 2 (see, for example, Saldin *et al.*, 2010 ▸).

However, in the presence of additional radiation fluctuations or their suppression due to poor spatial coherence or insufficient resolving power of the monochromator, this value may be higher or lower, respectively.

Lutman *et al.* (2012 ▸) suggested to treat the value of  as a quality factor of the potential reconstruction at a given frequency. In Fig. 18 ▸ we show how this value is affected upon introducing various detrimental effects.

## Appendix D Analogy with the Wiener–Khinchin theorem

One can see that the ROSA evaluation formula

is very similar to the formulation of the Wiener–Khinchin theorem

This theorem states that the statistical autocorrelation function of a *stationary* random process in the *time* domain and the *ensemble-averaged* spectral density, *i.e.* power spectrum, of that process forms a Fourier transform pair. The Wiener–Khinchin theorem plays a central role in Fourier-transform spectroscopy and allows one to calculate the radiation spectral density after directly measuring the amplitude autocorrelation function of the radiation in the time domain.

In contrast, we calculate the *square* of the statistical autocorrelation function of a *non-stationary* process in the *frequency* domain and carry out the Fourier transform to the time domain in order to obtain the reconstruction function *R*(*t*,ω).

Should the considered process be quasistationary, the function *R* would be factorizable, and the autocorrelation of its *ensemble-averaged* power profile could be reconstructed with equation (21). The latter is simply the combination of the autocorrelation theorem and the Wiener–Khinchin theorem applied in the opposite domain.

In Fourier-transform spectroscopy the time autocorrelation function Γ is obtained by carrying an average over time, as the investigated process is assumed to be ergodic. This averaging naturally leads to the loss of the dependence of the function Γ on time, so one obtains the statistical autocorrelation function of time delay Γ(Δ*t*) over which the Fourier transform is later performed.

In our case, the frequency autocorrelation function  is calculated via averaging over an ensemble of single-shot spectra. Such averaging is justified by the statistical properties of the SASE radiation, as discussed in Appendix *B* [App appb]. The dependence on both central frequency ω and frequency difference Δω remains, yielding additional information upon the Fourier transform over Δω.
